# Unusual Bilateral Rim Fracture in Femoroacetabular Impingement

**DOI:** 10.1155/2015/210827

**Published:** 2015-02-05

**Authors:** Claudio Rafols, Juan Edo Monckeberg, Jorge Numair

**Affiliations:** MEDS Clinical Sport Center, 8320000 Santiago, Chile

## Abstract

This is a report of one case of bilateral acetabular rim fracture in association with femoroacetabular impingement (FAI), which was treated with a hip arthroscopic procedure, performing a partial resection, a labral reinsertion, and a subsequential internal fixation with cannulated screws. Up to date, there are in the literature only two reports of rim fracture and “os acetabuli” in association with FAI. In the case we present, the pincer and cam resection were performed without complications; the technique used was published previously. With this technique the head of the screw lays hidden by the reattached labrum. We removed partially the fractured rim fragment and the internal fixation of the remaining portion was achieved with a screw. In the event of a complete resection of the fragment, it would have ended with a LCE angle of 18° and a high probability of hip instability. We believe that this bilateral case helps establish the efficacy and reproducibility of the technique described by Larson.

## 1. Introduction

Recently, Ganz et al. have identified in femoroacetabular impingement (FAI) a predominant cause of labral tear of the hip; this situation may be the result of an abnormal contact between the proximal femur and the acetabular rim [1]. Sometimes, in particular cases, a specific pincer type of femoroacetabular impingement can be associated with “os acetabuli” or with bone rim stress fracture [2, 3]. The nature of bone fragments about the acetabular rim is a reason for discussion; some groups have mentioned that these are remnants of the ossification nucleus and that the fragment can be seen on X-ray examination at the age of seven years and they may well disappear before 20 years of age. Other radiologists and clinicians have described these fragments as being part of the evolution of Perthes disease or an anatomical variation in congenital hip dysplasia [4, 5]. Epstein and Safran [3] have stated that the “os acetabular” and the rim stress fracture may be the result of an abnormal acetabular development or the continuous pincer impingement contact. In contrast, resection of large “os acetabuli” can lead to a structural instability of the joint [3].

This is a report of one case of bilateral acetabular rim fracture in association with FAI, which was treated with a hip arthroscopic procedure, performing a partial resection (pincer), a labral reinsertion, and a subsequential internal fixation with cannulated screws.

## 2. Case

**Figure 1:**
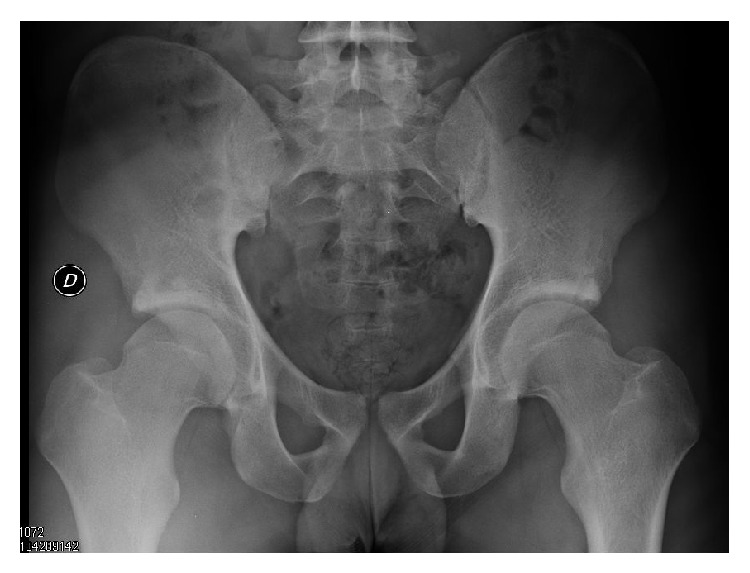
AP view of both hips showed a large superior rim fracture.

A 20-year-old competitive male soccer player, who presented with a two-year history of recurrent bilateral groin pain and stiffness, was admitted to our institution. Bilateral examination showed pain in hip flexion, adduction, and internal rotation (anterior impingement test); abduction and external rotation were normal. The patient had bilateral hip flexion up to 100° and internal rotation of 0°. Plain radiographs of the pelvis showed a well maintained joint space and a combined type FAI image. The alpha angles were 78° for the right side and 76° for the left side; the lateral centre edge (LCE) angle was 45° on the right and 47° on the left. Both hips showed a large superior rim fracture (Figure 1).

**Figure 2:**
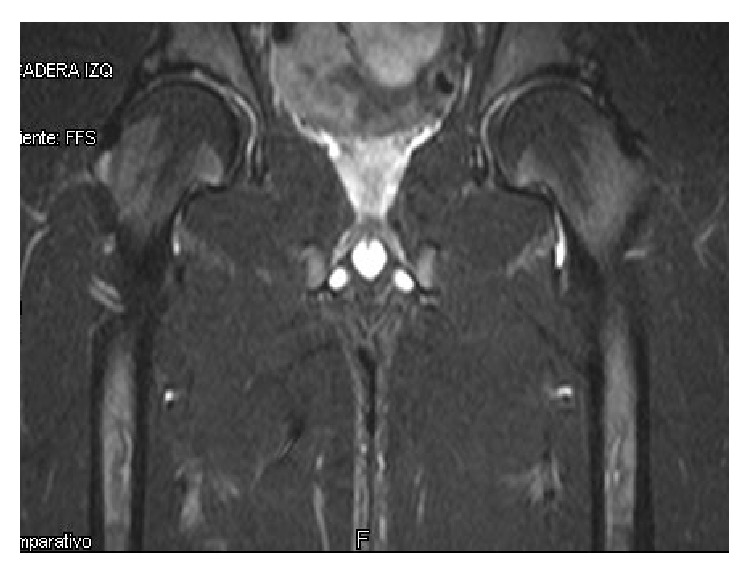
T2 magnetic resonance arthrogram showed FAI and an anterosuperior bilateral labral tear.

**Figure 3:**
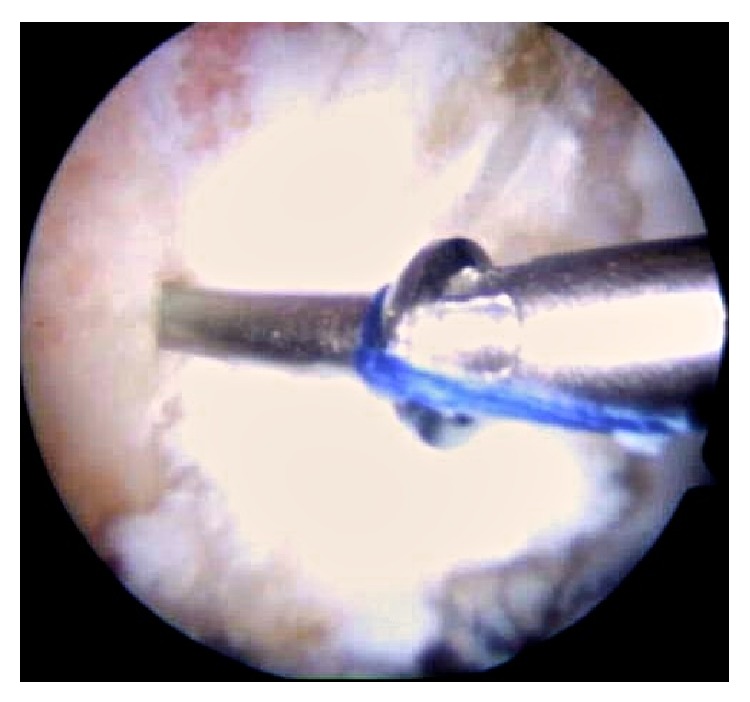
Under X-ray supervision, the remaining bone fragment was secured with an arthroscopic assisted 3.0 mm cannulated screw in both sides.

A magnetic resonance arthrogram showed FAI and an anterosuperior bilateral labral tear (Figure 2). A bilateral hip test with intra-articular lidocaine was positive (showing an intra-articular and labral pain). The patient was then treated with an arthroscopic procedure of each hip, with a three-month gap period in between them both. The out-in hip arthroscopy approach described by Sampson [6] was used in both surgical events. A labral tear associated with a large superior fracture of the bone rim was found; the cartilage at the junction with the labrum was intact in both hips. Pincer resection was performed with power instruments, which included about 30% of the fractured segment. This was performed under X-ray supervision and the remaining bone fragment was secured with an arthroscopic assisted 3.0 mm cannulated screw (Figure 3) in both sides. Finally, the labral fixation was secured with 3 translabral suture anchors and an arthroscopic cam resection of the femoral neck was performed. The final examination of the hip motion under arthroscopic vision probed no impingement at all.

The patient was restricted to foot flat weight bearing and two crutches for 6 weeks and progressed back into soccer at 4 months postoperatively. Physiotherapy was indicated starting the 5th day after surgery. At two-year follow-up, he had no pain during soccer or other athletic activities, the hip impingement signs were negative, and the range of motion of his hips had improved to 120° of flexion and 30° of internal rotation. His modified Harris Hip Score showed a variation from 81.3 to 100 points on the right and from 87.1 to 96.1 points on the left, and his visual analogue pain score decreased from 8 to 1.

**Figure 4:**
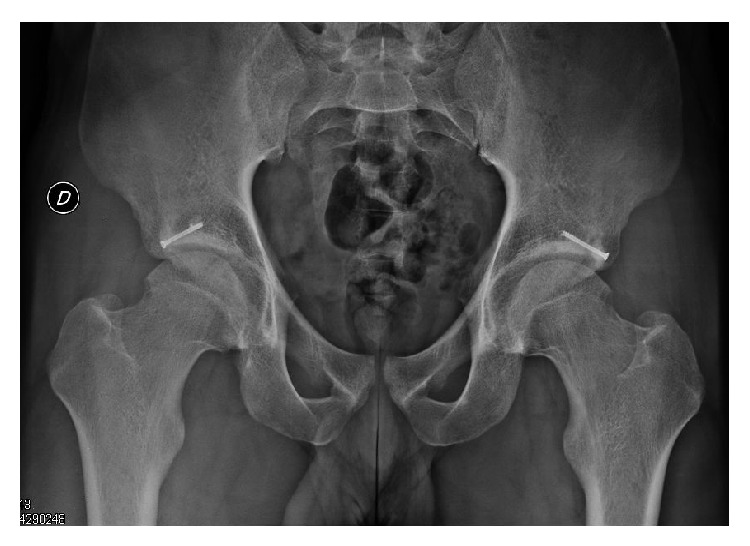
Pelvis AP radiographs at follow-up taken at* six months*  showed that the rim fracture healed. The LCE angle had decreased from 45° to 32° postoperatively in both hips and the hip joint space was still well maintained.

Radiographs at follow-up taken at* six months*  showed that the rim fracture healed. The LCE angle had decreased from 45° to 32° postoperatively in both hips and the hip joint space was still well maintained (Figure 4).

## 3. Discussion

Morphological abnormalities of the hip, particularly the proximal femur (cam) and the acetabulum (pincer), have been described as causes of femoroacetabular impingement. During joint motion and weight bearing in hips with FAI abnormal bony contact may occur, and soft tissue structures (cartilage and labrum) could fail [7]. It is also known that this disease has a variable presentation in between patients [8].

The literature indicates that arthroscopic management of FAI has shown improvements in its results when the indication is correctly achieved and the technique is properly performed [8–12]. It has been described in the presence of pincer that “os acetabuli” and rim fractures may present with a prevalence of 3.6% [13]. One hypothesis is that “os acetabuli” and rim fractures are the result of abnormal acetabular development or the consequence of stress fractures from repetitive contact of the femoral neck against the acetabular rim [3, 14], although these fragments can be completely excised, as part of the FAI correction procedure, and cause instability [3, 13, 14].

It is very important to maintain the stability of the hip; on one side, it is determined by an appropriate acetabular coverage of the femoral head, and, on the other side, the joint capsule and soft tissues play an important role as stabilizing structures; there are some published reports which show iatrogenic dislocations and subluxations after excessive arthroscopic rim resections [15–17]. The function of the “os acetabuli” or the bone rim in the acetabular coverage is not clear [18].

Up to date, there are in the literature only two reports of rim fracture and “os acetabuli” in association with FAI. The first is a report where Epstein and Safran [3] in 2009 reported one case of arthroscopic internal fixation of an unstable fracture of the acetabular rim after removal of the fibrocartilaginous junction and the other is a paper presented by Larson and Stone in 2011 [2] where they performed arthroscopic partial excision and internal fixation, both with excellent results at two years of follow-up.

In the case we present, the pincer and cam resection were performed without complications; the technique used was published by Larson and Giveans [11]. As previously described, the fixation was performed with cannulated screws, a method that does not interfere with the arthroscopic instruments; the insertion of the screw is achieved under direct arthroscopic vision and fluoroscopic guidance.

With this technique, the head of the screw lays hidden by the reattached labrum. Like in Larson's publication, we removed partially the fractured rim fragment and the internal fixation of the remaining portion was achieved with a screw. In the event of a complete resection of the fragment, it would have ended witha LCE angle of 18° and a high probability of hip instability.

In an attempt to promote healing and ossification of the fracture rim, drilling of the interface with a k-wire is recommended. We believe that correcting the hip impingement and fixing the acetabular fragment with an internal fixation eliminate the shearing forces due to FAI and create compression due to the screw, allowing the fragment to heal.

With regard to the surgery gap between both sides, this was because the need of partial load of the operated hip was required in 8 weeks. We believe that this bilateral case may help establish a therapeutic management, described by Larson and reproduced by these authors, and that will bring a little more scientific experience in this rare pathology.
